# Tradeoffs in Introduction Policies for the Anti-Tuberculosis Drug Bedaquiline: A Model-Based Analysis

**DOI:** 10.1371/journal.pmed.1002142

**Published:** 2016-10-11

**Authors:** Amber Kunkel, Frank G. Cobelens, Ted Cohen

**Affiliations:** 1 Department of Epidemiology, Harvard T.H. Chan School of Public Health, Boston, Massachusetts, United States of America; 2 Department of Epidemiology of Microbial Diseases, Yale School of Public Health, New Haven, Connecticut, United States of America; 3 Department of Global Health, Academic Medical Center, Amsterdam, Netherlands; 4 KNCV Tuberculosis Foundation, The Hague, Netherlands; University of California San Francisco, UNITED STATES

## Abstract

**Background:**

New drugs for the treatment of tuberculosis (TB) are becoming available for the first time in over 40 y. Optimal strategies for introducing these drugs have not yet been established. The objective of this study was to compare different strategies for introducing the new TB drug bedaquiline based on patients’ resistance patterns.

**Methods and Findings:**

We created a Markov decision model to follow a hypothetical cohort of multidrug-resistant (MDR) TB patients under different bedaquiline use strategies. The explored strategies included making bedaquiline available to all patients with MDR TB, restricting bedaquiline usage to patients with MDR plus additional resistance and withholding bedaquiline introduction completely. We compared these strategies according to life expectancy, risks of acquired resistance, and the expected number and health outcomes of secondary cases.

For our simulated cohort, the mean (2.5th, 97.5th percentile) life expectancy from time of initiation of MDR TB treatment at age 30 was 36.0 y (33.5, 38.7) assuming all patients with MDR TB received bedaquiline, 35.1 y (34.4, 35.8) assuming patients with pre-extensively drug-resistant (PreXDR) and extensively drug-resistant (XDR) TB received bedaquiline, and 34.9 y (34.6, 35.2) assuming only patients with XDR TB received bedaquiline. Although providing bedaquiline to all MDR patients resulted in the highest life expectancy for our initial cohort averaged across all parameter sets, for parameter sets in which bedaquiline conferred high risks of added mortality and only small reductions in median time to culture conversion, the optimal strategy would be to withhold use even from patients with the most extensive resistance. Across all parameter sets, the most liberal bedaquiline use strategies consistently increased the risk of bedaquiline resistance but decreased the risk of resistance to other MDR drugs. In almost all cases, more liberal bedaquiline use strategies reduced the expected number of secondary cases and resulting life years lost. The generalizability of our results is limited by the lack of available data about drug effects among individuals with HIV co-infection, drug interactions, and other sources of heterogeneity, as well as changing recommendations for MDR TB treatment.

**Conclusions:**

If mortality benefits can be empirically verified, our results provide support for expanding bedaquiline access to all patients with MDR TB. Such expansion could improve patients’ health, protect background MDR TB drugs, and decrease transmission, but would likely result in greater resistance to bedaquiline.

## Abstract 2

Amber Kunkel and colleagues present model-based analysis to identify optimal strategies for introducing the new anti-tuberculosis drug bedaquiline.

## Author Summary

**Why Was This Study Done?:**

- Bedaquiline is a new tuberculosis (TB) drug approved by the United States Food and Drug Administration in 2012 for patients with multidrug resistant (MDR) TB without other treatment options.
- Although the initial clinical trials of bedaquiline in combination with an optimized background regimen for MDR TB showed promising efficacy, one of these studies inexplicably had more deaths in the study group receiving bedaquiline.
- The individual and public health benefits of providing bedaquiline to different categories of TB patients are unclear.

**What Did the Researchers Do and Find?:**

- We used a mathematical decision model to simulate the potential effects of providing bedaquiline to different categories of TB patients based on their drug resistance patterns.
- We found that strategies that conservatively limit bedaquiline access to all but the most resistant patients would minimize risks of resistance to bedaquiline but maximize risks of resistance to important background drugs such as moxifloxacin.
- We predict that more liberal bedaquiline use strategies would lower transmission and improve health outcomes among secondary cases.
- We found that if bedaquiline safety and efficacy are assumed to be sufficiently high, the optimal strategy in terms of individual patient life expectancy would be to provide bedaquiline to all patients with MDR TB.

**What Do These Findings Mean?:**

- Researchers should prioritize collecting additional data to establish a mortality benefit of bedaquiline.
- If the safety of bedaquiline is confirmed, expanding bedaquiline access to all patients with MDR TB could improve patients’ health, prevent resistance to background MDR TB drugs, and decrease transmission, but would likely result in greater resistance to bedaquiline.

## Introduction

Only approximately 50% of the 111,000 people started on treatment for multidrug-resistant tuberculosis (MDR TB) in 2014 are likely to be successfully treated [1]. The remainder will experience high mortality, risk acquisition of extensively drug-resistant (XDR) TB, and may continue to infect others. New antibiotics have the potential to improve both prevention and treatment of highly drug resistant TB. Bedaquiline and delamanid recently became the first new drugs approved for TB treatment in over 40 y [2,3], and other promising drugs such as pretomanid are in development [4]. Effective drug use policies will be necessary to obtain maximal benefit from these new drugs while also managing risks of resistance.

Although clinical management of TB relies on strong multidrug regimens, the initial discovery and development of new TB drugs often occur in isolation. Optimizing multidrug regimens is complicated in both theory (e.g., by the number of drugs, limited data on drug efficacy and interactions, and the prevalence of existing resistance) and practice (e.g., by lack of access to patients’ full drug susceptibility profiles and limited opportunity for controlled trials) [5,6]. Thus, decisions about how best to introduce and combine new TB drugs have relied heavily on expert opinion. Limited guidance exists beyond common-sense strategies, such as never to add a single drug to a failing regimen, and broad considerations, such as the number of drugs and their side-effect profiles [5,7].

Here, we present a Markov decision model to begin formalizing a rational basis for decisions about drug introduction. Using the model, we outline the tradeoffs involved in deciding which patients should receive a new anti-TB drug, based on both their outcomes and those of their immediate contacts. We explore a continuum of policies ranging from most conservative (i.e., restricting the new drug entirely or for use only among the most highly resistant patients) to most liberal (i.e., allowing all patients with MDR TB to receive the new drug). Though the general framework of our analysis is broadly generalizable, we focus this paper specifically on the new TB drug bedaquiline. Bedaquiline was approved by the United States Food and Drug Administration in 2012 for use in MDR TB patients without other treatment options on the basis of its Phase IIb trial culture conversion results. However, concerns about resistance and a mortality imbalance observed in the pivotal Phase IIb trial have generated controversy about the appropriate role of this new drug [8–11]. A formal approach to assessing potential bedaquiline use strategies is therefore especially appropriate.

## Methods

This modeling study was based on previously published aggregate data and thus did not require ethical approval. To evaluate the impact and potential tradeoffs of different bedaquiline introduction strategies, we created a Markov decision model following a hypothetical cohort of patients initiating MDR TB treatment and their immediate contacts. A model description is provided below, with additional details available in S1 Appendix sections 2, 7, and 8.

### Population

Our assumed population was a cohort of European men initiating MDR TB treatment at age 30. All men were assumed to be bedaquiline susceptible at baseline and have either MDR TB without additional resistance (“MDR” from here), MDR TB with additional resistance to either at least one fluoroquinolone or at least one second-line injectable, but not both (“PreXDR”), or MDR TB with additional resistance to at least one fluoroquinolone and at least one second-line injectable (“XDR”). We assumed that 6.7% of patients initially had XDR TB, 26.2% of patients initially had PreXDR TB, and the remaining 67.1% of patients had MDR TB without additional resistance to the fluoroquinolones or second-line injectables, as observed in one published multi-country patient cohort [12].

### Health States and Transitions

**Fig 1:**
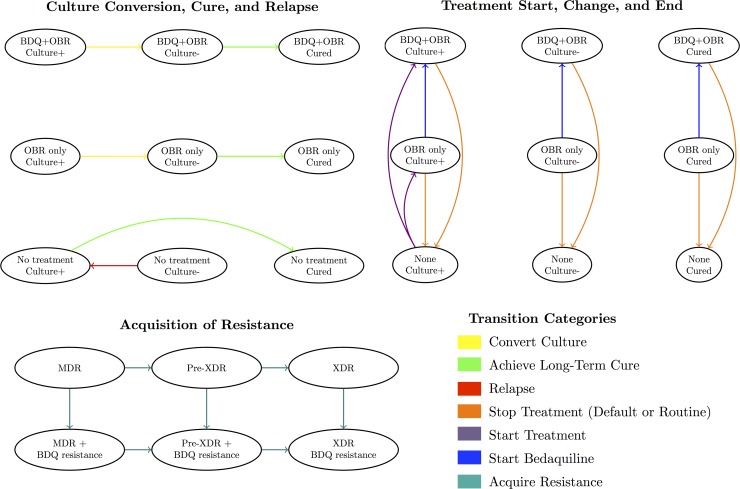
Overview of model health states and transitions. An individual’s health state at any given time reflects their culture status, treatment regimen, and resistance profile. See S1 Appendix section 8 for a complete list of states and transitions.

Fig 1 displays the categories of health states and transitions included in our model. Modeled health states were defined based on TB culture status (positive, negative, or stable cure), treatment regimen (optimized background regimen [OBR]; OBR plus bedaquiline; or no treatment), and resistance pattern (to bedaquiline and background drugs). Transitions between these states included culture conversion, relapse, routine or premature cessation of treatment, treatment re-initiation after cessation, regimen change, resistance acquisition, and death. We assumed that resistance was acquired in a stepwise fashion (i.e., to one drug at a time), and that patients could only relapse after treatment (i.e., culture conversions were only modeled if sustained through the end of treatment). We also assumed that TB-related mortality and acquired resistance rates applied only to patients who were culture-positive, and that some patients self-cured even in the absence of TB treatment.

### Treatment Strategies

We considered the following treatment strategies: withholding bedaquiline from all patients, providing bedaquiline to patients with XDR TB only, providing bedaquiline to patients with PreXDR or XDR TB, or providing bedaquiline to all patients with at least MDR TB. We did not allow treatment to differ based on bedaquiline resistance patterns, reflecting the current lack of a validated test with breakpoints defining clinically relevant bedaquiline resistance [5].

For the strategy in which all patients with MDR TB were eligible for bedaquiline, we assumed that all patients received bedaquiline from the beginning of treatment. For the more conservative strategies, we assumed a 13-wk average lag time after acquisition of or treatment initiation with the relevant resistance pattern to account for a delay in obtaining results of second-line drug susceptibility testing (DST). We compared these results to an analysis assuming no lag time, reflecting the potential impact of widespread rapid second-line DST availability.

### Outcomes

We considered mortality, resistance, and transmission outcomes. To assess mortality, we compared the average life expectancy from initiation of MDR TB treatment across the different bedaquiline use strategies, and to assess resistance, we recorded the number of patients who acquired particular resistance patterns under each treatment strategy. To assess transmission, we estimated the number of secondary cases as well as life years lost to secondary cases. The methodology used for these estimates is described below.

### Transmission to Secondary Cases

To assess transmission, we first calculated an approximate number of secondary cases infected by our initial cohort from initiation of MDR TB treatment as follows. We assumed that a single infectious, untreated, drug susceptible individual would infect others at a rate of ten infections per year, and that each infected individual had a 10% chance of progressing to active disease at some point in his or her lifetime [13–15]. We also allowed for varying transmission costs depending on the resistance pattern of the infecting patient, ranging from 0.5 for XDR to 0.7 for MDR in the absence of bedaquiline resistance [16–22]. To estimate the number of secondary cases produced per year by a single untreated, culture-positive case, we multiplied the infection rate by the progression probability and the applicable transmission cost. We reduced this value 5-fold for individuals receiving treatment, assuming that treatment would reduce infectiousness by a value similar to the relative infectiousness of smear-negative as compared to smear-positive TB [13,23,24]. We then converted these values into weekly infection rates and applied them to the individuals in our model based on their culture, resistance, and treatment status at each time step. Greater details and justification for the parameters used are provided in the parameter table in S1 Appendix section 7.

The life expectancy of each secondary case was calculated based on the resistance pattern of the index case at the time of the infection event, with background mortality rates reflecting those used for our initial cohort (i.e., assuming similar demographics to our initial cohort). Secondary cases were subjected to the same treatment strategy as the initial cohort. We assumed secondary cases had similar delays to detection as our initial cohort but were immediately recognized as MDR upon presentation to the health system. Detection of additional resistance was subject to similar delays as for the index patients. To calculate the expected number of life years lost to secondary cases under each treatment scenario, we combined these estimates of the life expectancy among secondary cases with our estimates of the number of secondary cases. These values are intended to be a first approximation of the transmission impact of differing bedaquiline use policies and do not capture the full range of MDR TB transmission dynamics, including within-household transmission and time to disease progression.

### Parameterization

Parameters describing TB natural history and outcomes in the absence of bedaquiline were taken from published cohorts, clinical trials, and meta-analyses [25–27]. These parameters were held fixed throughout our analysis. Parameters describing the effect of bedaquiline were derived from the bedaquiline pivotal trials [8,28] and more recent cohorts [3,29,30]. An overview of these studies is included in S1 Appendix section 1. Because only small numbers of patients receiving bedaquiline-containing regimens had completed treatment at the time of this analysis, we explored wide uniform ranges of values for key bedaquiline-associated parameters as described in Table 1. Additional mortality results based on triangular distributions are included in S1 Appendix section 4 and are qualitatively similar to the results from our uniform distributions included in the main text.

**Table 1:** Bedaquiline-associated parameter ranges.

Parameter	Distribution	References/Explanation
Default rate on bedaquiline (versus OBR)	Unif(-10%,+10%)	[31,32]; baseline chosen such that 17.3% of people with XDR on OBR default
Risk of relapse on bedaquiline (ratio to OBR)	Unif(0.4,1)	[27,28,33]; on OBR, the proportion who ever relapse is 4% with MDR, 8% with PreXDR, and 16% with XDR
Median time to culture conversion on bedaquiline (ratio to OBR)	Unif(0.4,1)	[8,28–30,34,35]; on OBR, median time to conversion is 13 wk for those MDR at baseline (18 for PreXDR, 26 for XDR)
Bedaquiline-associated mortality rate (addition to TB or background mortality)	Unif(0, 5 per 100 person-years) → Unif(0, 0.00096) weekly probability	[8]: 3 deaths in BDQ arm in overall treatment phase. 79 people assigned to BDQ, 50 completed treatment (~2 years); [29,30,36]
Risk of acquired bedaquiline resistance	Unif(0.1,0.5) for XDR 4x lower for PreXDR 16x lower for MDR	[8,25,28]
Risk of acquired resistance to background drugs on OBR (ratio to on bedaquiline)	Unif(1.05,8)	[8,25,28]; on OBR, 10.3% MDR acquire PreXDR, and 26% PreXDR acquire XDR
Transmission fitness of bedaquiline resistance (ratio to bedaquiline sensitive)	Unif(0.7,1)	Similar to other TB drugs [16–19]

### Calculation

All analyses were performed in TreeAge Pro 2015 R2.2. We assumed that transitions occurred on a discrete weekly basis, allowing us to capture potentially rapid changes in infectiousness, prognosis, and resistance patterns. From our bedaquiline-associated parameter ranges, we sampled 5,000 random parameter sets and for each estimated expected values for life expectancy, resistance acquisition patterns, and number and outcomes of secondary cases under each treatment scenario. We then calculated the average outcome for each strategy across all parameter sets, as well as the number of parameter sets for which each strategy was optimal (i.e., produced the maximum or minimum expected outcome across all strategies, as appropriate).

## Results

**Fig 2:**
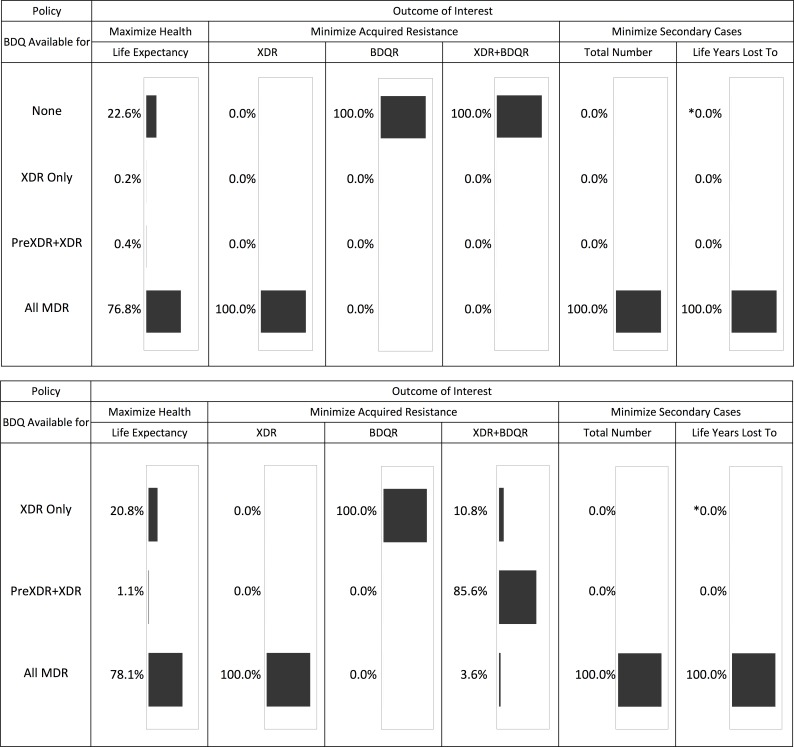
Optimal bedaquiline use strategy for different outcomes based on 5,000 simulation runs. The top half of the figure shows the results across all four potential bedaquiline use strategies. The bottom half shows results assuming bedaquiline is made available for at least some patients (i.e., no “none” strategy). The asterisk indicates that one simulation run resulted in this simulation being optimal. See tables for results on the magnitude of differences between strategies.

Fig 2 summarizes the optimal bedaquiline use strategies from each simulation for a range of mortality, resistance, and transmission outcomes. An overview of these results and additional analyses for each outcome are provided below.

### Life Expectancy

Providing bedaquiline to all patients with MDR TB maximized the life expectancy of our initial cohort in 76.8% of 5,000 simulations (Fig 2). In nearly all remaining simulations, the optimal strategy was to withhold bedaquiline from all patients, suggesting that the benefits of bedaquiline did not outweigh potential added mortality risks. Intermediate bedaquiline use strategies were optimal in fewer than 1% of simulations. Average life expectancy following the best strategy for each individual parameter set was 36.12 y (after MDR TB treatment initiation at age 30) compared to 34.67 y under the worst strategy for a difference of 1.45 y.

**Fig 3:**
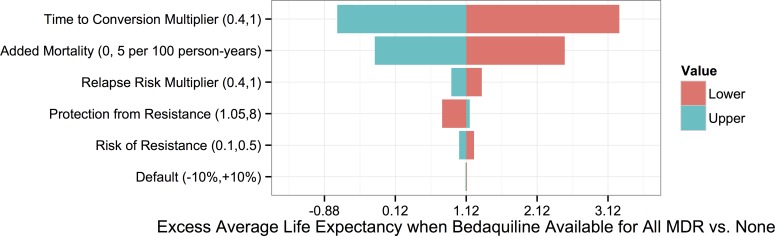
Tornado plot displaying how the potential improvement in average life expectancy that would result from use of bedaquiline for all patients with MDR TB versus no patients depends on the values of particular parameters. The *y*-axis displays each bedaquiline-associated parameter as well as its high and low values.

**Fig 4:**
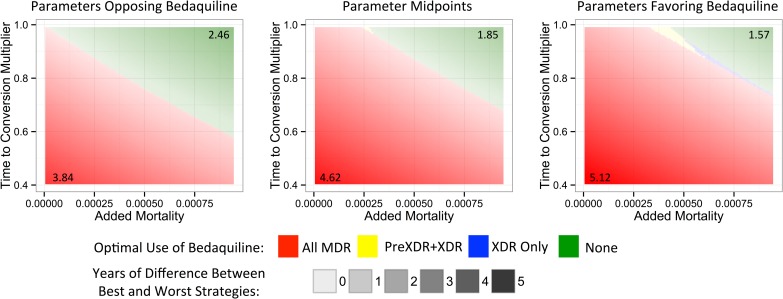
Heat maps showing regions in which each bedaquiline use strategy would be preferred. The *x-* and *y*-axes show the explored rates of (relative) median time to culture conversion and added mortality associated with bedaquiline use. Remaining parameters are fixed at their values that least favor bedaquiline (left), their midpoints (middle), and their values that most favor bedaquiline (right). Colors indicate the optimal bedaquiline use strategy, and shading indicates the magnitude of difference in average life expectancy between the best and worst strategies, with the corner values listed on the figure (in years). The PreXDR+XDR strategy is never selected in the left subplot, and the XDR strategy is never selected in the left or center subplots.

To understand which parameters were most responsible for the variation in life expectancy outcomes associated with each strategy, we first created a tornado plot (Fig 3) showing the impact of varying each parameter to its low and high values while keeping all other variables fixed at their midpoints. As shown in this figure, the most influential parameters are the rates of added mortality and culture conversion associated with bedaquiline. Fig 4 displays the impact of these two parameters on the optimal bedaquiline use strategy more directly, with the remaining parameters set to their midpoints as well as their extreme values that most favored and opposed use of bedaquiline in all patients. Providing bedaquiline to all patients is preferred when bedaquiline strongly reduces median time to culture conversion and has low added mortality risk, whereas withholding bedaquiline from all patients is preferred when bedaquiline has high mortality risks and a low impact on time to culture conversion. For example, when all other parameters are fixed at their midpoints, the “All MDR” strategy is preferred whenever bedaquiline reduces the median time to culture conversion compared to OBR only by at least 35%, regardless of the added mortality risk (within the range explored). Similarly, when all other parameters are fixed at their midpoints, the “All MDR” strategy is always preferred whenever the added mortality risk associated with bedaquiline is less than 0.00025 per week, or 1.3 excess deaths per 100 person-years, regardless of the effect of bedaquiline on time to culture conversion. Of note, the “All MDR” strategy is preferred for a majority of the combinations of culture conversion and added mortality, regardless of the values of all other parameters, highlighting the importance of prioritizing these values for future study.

Table 2 displays the effect of the DST methods available to detect PreXDR and XDR TB on life expectancy under the different bedaquiline use strategies. The rapid DST method, which shortens the lag time for eligible individuals to receive bedaquiline, increased the average life expectancy for both the “XDR only” and “PreXDR+XDR” strategies. Notably, the availability of rapid second-line DSTs also changes the proportion of times each strategy would be optimal in terms of life expectancy, with the “all MDR” scenario providing optimal life expectancy for 69.3% of scenarios, compared with 16.9% for “PreXDR+XDR” and 13.7% for “None.” The “XDR only” strategy was chosen only once out of 5,000 runs. These results suggest that widespread availability of rapid second-line DSTs could alter decisions about optimal bedaquiline use. However, the “all MDR” scenario was still the most frequently chosen, and its average life expectancy still exceeded those for the “PreXDR+XDR” as well as “XDR only” strategies, suggesting that the potential benefits of making bedaquiline available for all patients with MDR TB extend beyond simply shortening the time to bedaquiline initiation for patients with more extensive resistance. Additional information on the parameter space in which each strategy would be preferred if rapid second-line DSTs were available is provided in S1 Appendix section 5.

**Table 2:** Life expectancy by DST method.

	Life Expectancy when BDQ Available for			
DST Method	All MDR	PreXDR+XDR	XDR Only	None
Conventional (Baseline)	36.0 (33.5, 38.7)	35.1 (34.4, 35.8)	34.9 (34.6, 35.2)	34.8
Rapid		35.5 (34.5, 36.7)	35.0 (34.6, 35.5)	

Life expectancy from initiation of MDR TB treatment at age 30 comparing bedaquiline (BDQ) use strategies under our baseline scenario (conventional DST to identify PreXDR and XDR cases) and a scenario with rapid DST for fluoroquinolones and injectables. Results are given as simulation mean (2.5 percentile, 97.5 percentile).

### Acquired Resistance

Fig 2 and Table 3 show the impact of different drug use strategies on acquired resistance to the new and existing drugs in our initial cohort. The best strategy to avoid resistance to bedaquiline was to strictly constrain bedaquiline availability. The simulation mean percentage of people acquiring resistance to bedaquiline was 5.88% (2.5th percentile 2.18%, 97.5th percentile 9.45%) in the scenario providing bedaquiline to all patients with MDR TB, compared with 3.50% (1.30%, 5.62%) when restricting bedaquiline for patients with XDR TB only. However, expanding bedaquiline availability is predicted to reduce the rate of acquired XDR TB by providing additional protection to the existing drugs. The percentage of people acquiring XDR TB was 2.56% (1.09%, 7.68%) in the scenario providing bedaquiline to all patients with MDR TB, compared with 9.82% (no variability, as non-bedaquiline parameters are assumed fixed) when restricting bedaquiline for patients with XDR TB only.

**Table 3:** Percentage of the initial cohort acquiring different resistance patterns.

	BDQ Available for			
% Acquiring	All MDR	PreXDR+XDR	XDR Only	None
BDQR	5.88 (2.18, 9.45)	3.91 (1.44, 6.29)	3.50 (1.30, 5.62)	0
PreXDR	2.50 (1.16, 6.43)	7.66	7.66	7.66
PreXDR+BDQR	1.93 (0.39, 3.69)	1.00 (0.16, 1.99)	0	0
XDR	2.56 (1.09, 7.68)	6.59 (5.84, 8.94)	9.82	9.82
XDR+BDQR	3.44 (1.29, 6.15)	3.20 (1.20, 5.23)	3.50 (1.65,5.62)	0

We only count patients who did not begin with the listed resistance pattern (e.g., patients who are initially XDR may be counted as acquiring “XDR+BDQR” but not “XDR”). Resistance patterns that are unspecified may have any value (e.g., “BDQR” identifies resistance to bedaquiline in combination with any pattern of OBR resistance). Gray shading indicates values that are necessarily the same as if bedaquiline were not available for any patients (“None”). Results are given as simulation mean (2.5th percentile, 97.5th percentile).

When we only consider scenarios in which at least some patients are eligible for bedaquiline, complete resistance to the new and existing drugs (XDR+bedaquiline resistance [BDQR]) was minimized most often by the intermediate strategy of providing bedaquiline to patients with PreXDR and XDR TB only. However, the “XDR only” strategy is preferred in 10.8% of the 5,000 simulation runs and the “all MDR” strategy in 3.6% of runs, indicating that the optimal decision for this outcome is parameter-dependent. This pattern reflects the differential effects of the bedaquiline use strategies on patients with different initial resistance patterns (see S1 Appendix section 6). For many (though not all) parameter sets, providing bedaquiline to all patients with MDR TB minimized the number of cases of acquired XDR+BDQR among patients with initial MDR or PreXDR TB, but maximized the number of cases of acquired XDR+BDQR among patients with initial XDR TB. However, the absolute differences in the number of cases of acquired XDR+BDQR across scenarios are small when bedaquiline is provided to at least some categories of patients, indicating that the costs of making a suboptimal decision with respect to this variable may be limited. The results are sensitive, however, to assumptions about time to treatment initiation; if we assume rapid second-line DSTs are available, providing bedaquiline to all patients with MDR TB is the most frequently preferred strategy (see S1 Appendix section 6).

### Secondary Cases

As shown in Table 4, the total number of secondary cases produced from the time of MDR TB treatment initiation was less than one per person across all treatment strategies, indicating non-sustainable transmission in the population from the point of appropriate treatment initiation. This number was higher but remained below one if we assumed individuals were initially untreated, reflecting the high mortality rate and lack of diagnostic delay in our model. Making bedaquiline available to all patients with MDR TB was the preferred strategy to minimize the number of secondary cases for all 5,000 simulation parameter sets and the years of life lost amongst secondary cases for all but one (Fig 2).

**Table 4:** Impact of different bedaquiline use strategies on the number and health outcomes of secondary TB cases.

	BDQ Available for			
Outcome per 100 Initial Patients	All MDR	PreXDR+XDR	XDR Only	None
Number of Secondary Cases	14 (10, 17)	17 (16, 18)	18 (18, 19)	19
Life Years Lost to Secondary Cases	243 (164, 317)	315 (290, 336)	333 (320, 343)	346

Results are given as simulation mean (2.5 percentile, 97.5 percentile).

## Discussion

New anti-TB drugs such as bedaquiline hold much promise to reduce morbidity and mortality associated with drug resistance. In this paper, we performed a decision analysis to explore the potential impact of different bedaquiline use strategies on a range of individual and public health outcomes. Different strategies may be preferred based on the outcome of primary interest (e.g., minimize resistance, minimize years of life lost), illustrating the tradeoffs involved in decision-making for the introduction of new antibiotics.

Drugs for which the risk of mortality due to adverse events exceeds expected reductions in mortality should not be used regardless of their potential public health benefits. Our model most often preferred providing bedaquiline to all patients with MDR TB for parameter sets in which bedaquiline introduced only a small added risk of mortality and substantially decreased the median time to culture conversion, and favored withholding bedaquiline from all patients when it was associated with high mortality and small declines in time to culture conversion. The frequency with which each of these parameters was preferred reflects the shape of our assumed uncertainty distributions, which we chose to be conservative estimates of the impact of bedaquiline; modifying these assumptions changes the quantitative results, but not the finding that in some cases withholding bedaquiline from all patients would be the preferred strategy (see S1 Appendix section 4). These results demonstrate the vital importance of continued research into bedaquiline safety and efficacy and support prioritizing additional safety data over secondary concerns such as the risk of acquired resistance. Although a model such as this can support such research prioritization, decisions about safety of bedaquiline must ultimately be based on data from real patients. Thus far, interim cohort analyses of patients receiving bedaquiline outside of trial settings have not identified excess bedaquiline-associated mortality [29,30]; however, continued data from compassionate use programs and, in particular, phase III trial results are needed to verify that the unexplained mortality imbalance of the pivotal phase IIb trial was not drug related.

Antibiotic introduction strategies may affect rates of acquired resistance to the new drug, existing drugs, or both. In general, we would expect more expansive access to a new drug to promote resistance to the new drug while preventing resistance to existing drugs. These expectations are reflected in our results. Acquired bedaquiline resistance occurred most often under the most liberal bedaquiline use policy (providing bedaquiline to all patients with MDR TB); however, this same policy was most effective at preventing new cases of PreXDR and XDR TB. The effects of expanding access to a new drug on composite resistance to new and existing drugs are less clear-cut. When considering only strategies providing bedaquiline to at least some categories of patients, the majority of our simulations predicted an intermediate strategy targeting bedaquiline to patients with PreXDR and XDR TB only to minimize the combination of XDR plus BDQR. However, both the “All MDR” and “XDR only” strategies were preferred for some combinations of parameter values, and differences in the proportions of people acquiring XDR+BDQR across different strategies were small. Because tuberculosis antibiotic resistance cannot be horizontally transferred, the spread of bedaquiline resistance to other patient cohorts is restricted to patients directly infected with bedaquiline-resistant bacteria. As such, although future spread of bedaquiline resistance will limit its benefits in terms of both patient health and protection for other drugs, we do not expect bedaquiline resistance to appear except in the context of background resistance patterns to which it is already being applied.

For this paper, we limited our assessment of future transmission of TB and drug resistance to the second generation of infected patients. We found that, for all but one of the 5,000 parameter sets tested, making bedaquiline available to all patients with MDR TB would minimize the total number of and expected number of life years lost to secondary cases. This relationship can be explained by the correlation between severe and highly infectious disease within our model. For diseases and treatments for which this assumption does not hold, associations may appear in the opposite direction [37]. Future drug development and policy changes may also affect the relationship between new drug use strategies and outcomes among potential secondary cases. Bedaquiline use strategies chosen now could alter the effectiveness of potential future TB regimens incorporating both bedaquiline (e.g., the NC-005 trial of bedaquiline, pyrazinamide, and pretomanid) and background drugs such as pyrazinamide and the fluoroquinolones (e.g., the STAND trial of pretomanid, moxifloxacin, and pyrazinamide) [3]. Of course, the desire to be prepared for the range of outcomes that could result from these trials must be weighed against the need to provide the best available care to patients presenting today. A full modeling analysis of these costs and benefits would require a transmission dynamic structure not included here.

This study has several limitations. We have not explored the full range of potential bedaquiline use strategies, e.g., as an early drug substitution method to prevent hearing loss during MDR TB treatment. For simplicity, we held the natural history and treatment parameters unrelated to bedaquiline fixed throughout our analysis, which does not reflect the potential uncertainty and heterogeneity in these parameters. Many of these estimates were based on large meta-analyses with data from multiple countries, allowing us to average over but not fully address the variability expected, e.g., in settings with standardized versus individualized treatment regimes. We assumed that our initial cohort was comprised of 30-year-old European men, which may differ from the target population of bedaquiline in many settings; however, as this assumption was used only in defining background mortality rates, it is most likely to affect the magnitude rather than the direction of the observed effects. Similarly, the effects of our particular background distribution of resistance are likely mitigated by the range of explored scenarios, which incrementally account for expanded access of bedaquiline to patients with XDR, then PreXDR+XDR, and finally all MDR. Changing the HIV status of this cohort could have greater effects if bedaquiline is found to have differential impact on HIV-positive and HIV-negative individuals. Similarly, we may see differential effects of bedaquiline if the background regimen varies substantially from the data on which our model was based, as in the STREAM II trial of shorter MDR regimens [3]. This limitation is especially relevant given the new World Health Organization guidelines that support the use of shortened MDR regimens for patients without anticipated second-line resistance, though we expect the differences between our PreXDR, XDR, and No Bedaquiline strategies to remain unchanged under this new policy [38]. Finally, we assumed that the efficacy of the background regimen did not differ depending on bedaquiline use; however, this may not be the case if it is necessary to modify the background regimen to avoid providing bedaquiline in combination with other QT-interval prolonging drugs.

Our results support the prioritization of verifying a mortality benefit of bedaquiline for patients with MDR TB. If such a benefit can be verified, they may provide support for expanded access of bedaquiline beyond the strict qualifications of compassionate use programs to all patients with MDR TB, particularly in settings where rapid second-line drug susceptibility testing is unavailable. Policymakers considering such expanded use should weigh the benefits of extending access to bedaquiline for all MDR TB patients seen in this analysis (including lower proportions of people acquiring resistance to background drugs and decreased onward transmission) against its potential drawbacks (including increased resistance to bedaquiline, as explored here, and the need to change the background regimen to avoid combining multiple QT-prolonging drugs, which we have not addressed).

## Supporting Information

S1 Appendix Additional model details and results. (PDF)

S1 Model TreeAge model used to calculate life expectancy and secondary case outcomes. (TREX)

S2 Model TreeAge model used to calculate resistance outcomes. (TREX)

## Abbreviations

BDQR: bedaquiline resistance
DST: drug susceptibility testing
MDR: multidrug-resistant
OBR: optimized background regimen
PreXDR: pre-extensively drug resistant
TB: tuberculosis
XDR: extensively drug resistant
