# Supplementary material for: Tradeoffs in Introduction Policies for the Anti-Tuberculosis Drug Bedaquiline: A Model-Based Analysis
Source: PLoS Med. 2016 Oct 11;13(10):e1002142. doi: 10.1371/journal.pmed.1002142 (PMC5058480; doi:10.1371/journal.pmed.1002142)
Supplement: S1 Appendix — (PDF) [file pmed.1002142.s001.pdf]

Supplementary Appendix for Tradeoffs in Introduction Policies for the Anti-Tuberculosis Drug Bedaquiline: A Model Based Analysis

Amber Kunkel, Frank G. Cobelens, Ted Cohen

Contents

1. Summary of included studies involving bedaquiline (p.1)
2. Additional calculation details (p.3)
3. Value of Information analysis (p.7)
4. Sensitivity to distributional assumptions (p.7)
5. Mortality sensitivity analysis, rapid second-line DSTs (p.8)
6. Additional analyses of XDR plus bedaquiline resistance (p.9)
7. Table of fixed parameters (p.10)
8. Complete list of model transitions (p.16)
9. References (p.25)

SUMMARY OF INCLUDED STUDIES INVOLVING BEDAQUILINE

The following list describes the clinical trial and cohort data on bedaquiline safety and efficacy that were used to inform this analysis. To our knowledge, this was at the time of our analysis an exhaustive list of the published data available on patients receiving bedaquiline for a full 6 months.

1

Title: Multidrug-resistant tuberculosis and culture conversion with bedaquiline

Authors: Diacon AH, Pym A, Grobusch MP, de los Rios JM, Gotuzzo E, Vasilyeva I et al.

Setting: Brazil, India, Latvia, Peru, the Philippines, Russia, South Africa, and Thailand

Study Design: Placebo-controlled clinical trial (bedaquiline or placebo added to optimized background regimen)

Number of Patients:

With 6 Months of Follow-Up: 66 (Modified Intention-to-Treat, assigned to bedaquiline)

With 24 Months of Follow-Up: 66 (Modified Intention-to-Treat, assigned to bedaquiline)

Overview:

As a placebo-controlled clinical trial, this study represents the highest quality of evidence available so far. Bedaquiline was found to reduce the median time to culture conversion to 83 days, compared with 125 days with placebo. Ten patients (of 79) in the bedaquiline group died during follow-up, compared with 2 (of 81) in the placebo group (P=0.02). None of the deaths were thought to be related to the study drugs, based on the judgment of a blinded investigator.

2

Title: Bedaquiline in the treatment of multidrug- and extensively drug-resistant tuberculosis

Authors: Pym AS, Diacon AH, Tang SJ, Conradie F, Danilovits M, Chuchottaworn C et al.

Setting: China, South Korea, the Philippines, Thailand, Estonia, Latvia, Russia, Turkey, Ukraine, Peru, and South Africa

Study Design: Open-label, single-arm trial

Number of Patients:

With 6 Months of Follow-Up: 205 (Modified Intention-to-Treat group)

With 24 Months of Follow-Up: 205 (Modified Intention-to-Treat group)

Overview:

This study contains the largest published cohort of patients receiving treatment with bedaquiline available at the time of our analysis. Patients were followed through 120 weeks. 16 deaths (6.9% of 233 patients, a lower proportion than that observed in Diacon et al) occurred during follow-up. The median time to culture conversion was 84 days. Although these results appear to represent an improvement over historical MDR TB cohorts, the trial setting and lack of a control group may limit the generalizability of these findings.

3

Title: Compassionate use of bedaquiline for the treatment of multidrug-resistant and extensively drug-resistant tuberculosis: interim analysis of a French cohort

Authors: Guglielmetti L, Le D D, Jachym M, Henry B, Martin D, Caumes E et al.

Setting: France

Study Design: Cohort of patients receiving bedaquiline

Number of Patients:

With 6 Months of Follow-Up: 35

With 24 Months of Follow-Up: None reported

Overview:

This study provides one of the earliest looks at bedaquiline used under programmatic conditions. The observed culture conversion rate was 97% after 6 months of therapy. Bedaquiline was frequently combined with linezolid and either a fluoroquinolone or second-line injectable. The results of this study are limited by its short duration of follow-up and lack of a control arm.

4

Title: Treatment of drug-resistant tuberculosis with bedaquiline in a high HIV prevalence setting: an interim cohort analysis

Authors: Ndjeka N, Conradie F, Schnippel K, Hughes J, Bantubani N, Ferreira H et al.

Setting: South Africa

Study Design: Cohort of patients receiving bedaquiline

Number of Patients:

With 6 Months of Follow-Up: 63

With 24 Months of Follow-Up: None reported

Overview:

This study contains the earliest published data on programmatic use of bedaquiline in a setting with high HIV prevalence. 76% of patients who completed six months of follow up either achieved culture conversion or remained culture negative. The authors highlighted the contrast between these results and those of the

2012 South Africa NTP MDR-TB cohort, in which culture conversion was only 56% at 6 months, but noted that other factors, such as expanded availability of Xpert MTB/RIF and linezolid, could explain some of the observed improvements. The results of this study, as for Guglielmetti et al., are limited by its short duration of follow-up and lack of a control arm.

5

Title: New developments in the treatment of drug-resistant tuberculosis: clinical utility of bedaquiline and delamanid (referencing presentation entitled: Armenian experience on treatment of XDR and pre-XDR patients with new drugs under compassionate use program)

Authors: Brigden G, Hewison C, Varaine F (referencing presentation by Yeghiazaryan L).

Setting: Armenia

Study Design: Cohort of patients receiving bedaquiline

Number of Patients:

With 6 Months of Follow-Up: 32

With 24 Months of Follow-Up: None reported

Overview:

This review paper included data presented at the 2015 Tuberculosis Symposium Eastern Europe and Central Asia on the Armenian experience on treatment of XDR and pre-XDR patients with new drugs under compassionate use program (available at: http://www.tb-symposium.org/2015/documents/en/presentations/Lusine_Yeghiazaryan_BDQ_practical_use_eng.pdf). 84% of patients who were culture positive at the time of treatment initiation had converted by 6 months. This study also had short follow-up and no control arm.

ADDITIONAL CALCULATION DETAILS

General Calculation Principles

Accounting for conditional probabilities

In constructing the model, the potential events for each week were modeled in sequence. For each weekly cycle we gave priority to events in an order that reflected the way they would be recorded as treatment outcomes. For example, for people receiving treatment we first we recorded all deaths. Those people who did not die could end treatment routinely, or if not they could end treatment prematurely (default). We assumed only patients who remained on treatment could have culture converted, and only those who did not culture convert could have acquired resistance (to at most one drug per week). To account for ordering, we input the probability of each event conditional on not experiencing any of the events earlier in the calculation sequence that week.

Examples:

- Weekly probability of culture conversion - input conditional on not dying, defaulting, or finishing treatment that week
- Weekly probability of stopping treatment routinely - conditional on not dying that week
- Weekly probability of default - conditional on not dying or stopping treatment routinely that week

Converting rates to weekly probabilities

To convert rates to weekly probabilities (p), we first converted them to rates per week. We then used the following formula:

$$p = 1 - exp(-rate)$$

Examples:

- Added mortality BDQ (rate 5 per 100 person-years → rate 5 per 5200 person-weeks = 0.00096 per person-week → weekly probability 1-exp(-0.00096)=0.00096)

Resistance and relapse probabilities

To convert the probability of acquiring resistance prior to death, conversion, default, or stopping treatment (Q) into weekly probabilities, we used the following equation with the weekly probabilities of each event (p):

$$Q_{res} = \frac{p_{res}}{p_{die} + p_{default} + p_{stop} + p_{convert} + p_{res}}$$

Examples:

- Probability acquired BDQ resistance given XDR
- Probability acquired XDR given pre-XDR, not on BDQ
- All other acquired resistance parameters (but may include additional considerations below)

A similar equation was used to relate the weekly cure rate to the probability of moving from "culture negative" (high risk of relapse immediately after treatment) to "stable cure" prior to death, default, or stopping treatment.

Median time to culture conversion

Based on our literature review, we estimated the median time to culture conversion (if no one had died or stopped treatment) to be approximately 13 weeks for people initially MDR, 18 weeks for people initially pre-XDR, and 26 weeks for people initially XDR. We used TreeAge to estimate a weekly probability of conversion based on these targets and our fixed weekly probabilities of acquiring pre-XDR and XDR TB.

To simplify our sensitivity analyses, we chose to consider the effect of BDQ on the median time to culture conversion if no one had died, stopped treatment, or acquired resistance. We did this by calculating the weekly probabilities of conversion from above to the median time of culture conversion if no one had died, stopped treatment, or acquired resistance. Our bedaquiline multiplier was then applied to these values.

We converted median time to event parameters (in weeks) to weekly probabilities using the geometric distribution:

$$P = 1 - 2^{-1/M}$$

Probability acquiring pre-XDR without BDQ

From the literature, we estimated that the probability of acquiring resistance to any fluoroquinolone given initial MDR was approximately 0.065, and that the probability of acquiring resistance to any second-line

injectable was similar. We also determined that the risk of XDR given pre-XDR was approximately 0.26. Note the informal notation: $P(PreXDR|MDR)$ is the probability of developing at least PreXDR for an individual who is initially MDR, and not receiving bedaquiline.

$$P(PreXDR|MDR) = P(FQR|MDR) + P(2LIR|MDR) - P(XDR|MDR)$$
$$P(PreXDR|MDR) = P(FQR|MDR) + P(2LIR|MDR) - P(PreXDR|MDR)P(XDR|PreXDR)$$
$$P(PreXDR|MDR) = .065 * 2 - 0.26 * P(PreXDR|MDR)$$
$$P(PreXDR|MDR) = 0.103$$

Probability acquiring pre-XDR with BDQ

This is similar to the section above, except we also need to account for the potential to acquire resistance to bedaquiline. Note the informal notation: $P(PreXDR|MDR)$ is the probability of developing at least PreXDR for an individual who is initially MDR (and BDQ sensitive), on bedaquiline.

$$P(PreXDR|MDR) = P(FQR|MDR) + P(2LIR|MDR) - P(XDR|MDR)$$
$$P(PreXDR|MDR) = P(FQR|MDR) + P(2LIR|MDR) - P(PreXDR|MDR)P(XDR|develop\,PreXDR)$$

The issue here is that $P(XDR|develop\,PreXDR)$ depends on whether the individual already has resistance to BDQ at the time they become PreXDR or not.

$$P(XDR|develop\,PreXDR) = P(already\,BDQR|develop\,PreXDR)P(XDR|PreXDR, BDQR)$$
$$+ (1 - P(already\,BDQR|develop\,PreXDR))\,P(XDR|PreXDR, BDQS)$$

To solve for $P(already\,BDQR|develop\,PreXDR)$, we can use our knowledge of the risk of resistance to bedaquiline for people who are initially MDR vs preXDR.

$$P(BDQR|MDR) = P(BDQR\,prior\,to/without\,PreXDR)$$
$$+ (1 - P(already\,BDQR|develop\,PreXDR))\,P(PreXDR|BDQS, MDR)P(BDQR|PreXDR, BDQS)$$

$$P(already\,BDQR|develop\,PreXDR) = \frac{P(BDQR\,prior\,to/without\,PreXDR)P(PreXDR|MDR, BDQR)}{P(PreXDR|MDR)}$$

Let X be the probability of developing bedaquiline resistance either prior to or without developing pre-XDR for people initially MDR on BDQ. Let Y be the probability of ever developing pre-XDR for people initially MDR on BDQ.

Let Q_{fm} be the probability of ever developing FQ resistance given MDR on BDQ. Let Q_{xpb} be the probability of ever developing XDR given pre-XDR and BDQ resistance. Let Q_{xp} be the probability of ever developing XDR given pre-XDR and BDQ sensitivity. Let Q_{bm} be the probability of developing bedaquiline resistance given initially MDR and BDQ sensitive. Let Q_{bp} be the probability of developing bedaquiline resistance given initially pre-XDR and BDQ sensitive. Let Q_{pmb} be the probability of developing pre-XDR given initially MDR and bedaquiline resistant.

$$Y = 2Q_{fm} - Y\left(\frac{XQ_{xpb}Q_{pmb}}{Y} + (1 - \frac{XQ_{pmb}}{Y})Q_{xp}\right)$$

$$Q_{bm} = X + (1 - \frac{XQ_{pmb}}{Y})YQ_{bp}$$

Solving the first equation for X:

$$Q_{bm} - YQ_{bp} = X - XQ_{pmb}Q_{bp}$$

$$Q_{bm} - YQ_{bp} = X(1 - Q_{pmb}Q_{bp})$$

$$\frac{Q_{bm} - YQ_{bp}}{1 - Q_{pmb}Q_{bp}} = X$$

Substituting into the first equation:

$$Y = 2Q_{fm} - Y\left(\frac{XQ_{xpb}Q_{pmb}}{Y} + (1 - \frac{XQ_{pmb}}{Y})Q_{xp}\right)$$

$$Y = 2Q_{fm} - XQ_{xpb}Q_{pmb} - YQ_{xp} + XQ_{xp}Q_{pmb}$$

$$Y = 2Q_{fm} - XQ_{pmb}(Q_{xpb} - Q_{xp}) - YQ_{xp}$$

$$Y = 2Q_{fm} - \frac{Q_{bm} - YQ_{bp}}{1 - Q_{pmb}Q_{bp}}Q_{pmb}(Q_{xpb} - Q_{xp}) - YQ_{xp}$$

We used Matlab's symbolic toolbox to solve this equation for Y. We checked this equation by verifying that our TreeAge model gave similar results for X and the probability of developing XDR given initially MDR and BDQ sensitive for a typical parameter set.

Probability acquiring XDR or BDQ resistance - from pre-XDR receiving BDQ

People can develop XDR without or prior to developing BDQ resistance, or they can acquire BDQ resistance first, increasing their chances of developing XDR. Similarly people can develop BDQ resistance without, prior to, or after developing XDR.

Let X be the weekly probability of developing BDQ resistance given pre-XDR. Let Y be the weekly probability of developing XDR given pre-XDR. Let A be the weekly probability of any other possible event (probability of dying or defaulting or finishing treatment or culture converting).

Let Q_{bp} be the probability of ever developing BDQ resistance starting pre-XDR, on BDQ. Let Q_{xp} be the probability of ever developing XDR starting pre-XDR, BDQ-sensitive, on BDQ. Let Q_{bx} be the probability of ever developing BDQ resistance starting XDR. Let Q_{xpb} be the proportion of people with pre-XDR and BDQ resistance who acquire XDR prior to death, default, finishing treatment, or culture conversion.

We can then use the following equations to solve for X and Y.

$$Q_{bp} = \frac{X}{X + Y + A} + Q_{bx}\frac{Y}{X + Y + A}$$

$$Q_{xp} = \frac{Y}{X + Y + A} + Q_{xbp}\frac{X}{X + Y + A}$$

Therefore

$$Q_{bp}(X + Y + A) = X + Q_{bx}Y$$

$$Q_{xp}(X + Y + A) = Y + Q_{xbp}X$$

Solving for X first:

$$(Q_{bp}) - 1)X = Q_{bx}Y - Q_{bp}(Y + A)$$
$$X = \frac{(Q_{bx} - Q_{bp})Y - Q_{bp}A}{Q_{bp} - 1}$$

Plugging into the equation for Y:

$$Q_{xp}(X + Y + A) = Y + Q_{xbp}X$$
$$(Q_{xp} - Q_{xbp})X + Q_{xp}A = Y - Q_{xp}Y$$
$$(Q_{xp} - Q_{xbp})\left(\frac{(Q_{bx} - Q_{bp})Y - Q_{bp}A}{Q_{bp} - 1}\right) + Q_{xp}A = Y - Q_{xp}Y$$
$$Q_{xp}A - (Q_{xp} - Q_{xbp})\left(\frac{Q_{bp}A}{Q_{bp} - 1}\right) = Y - Q_{xp}Y - (Q_{xp} - Q_{xbp})\left(\frac{(Q_{bx} - Q_{bp})Y}{Q_{bp} - 1}\right)$$
$$Q_{xp}A - \frac{(Q_{xp} - Q_{xbp})Q_{bp}A}{Q_{bp} - 1} = Y\left(1 - Q_{xp} - \frac{(Q_{xp} - Q_{xbp})(Q_{bx} - Q_{bp})}{Q_{bp} - 1}\right)$$
$$Y = \frac{Q_{xp}A(Q_{bp} - 1) - (Q_{xp} - Q_{xbp})Q_{bp}A}{(Q_{bp} - 1)(1 - Q_{xp}) - (Q_{xp} - Q_{xbp})(Q_{bx} - Q_{bp})}$$

VALUE OF INFORMATION ANALYSIS

We additionally conducted a Value of Information analysis to support our assessment of the parameters with greatest influence on our life expectancy outcomes (see Figures 3 and 4 in the Main Text). We first calculated the expected value of perfect information, or the additional life expectancy that one would expect to gain on average if there was no uncertainty in our bedaquiline-related parameters, based on 5,000 random parameter samples. We then calculated the expected value of partial perfect information separately for each of the bedaquiline parameters, sampling 200 values in the outer loop of the parameter of interest and 1000 values in the inner loop of the remaining parameters.

The results of our value of information analysis are provided in Table 1. The expected value of perfect information for all parameters is 0153 life years (per patient). The expected value of partial perfect information was 0 for all parameters except the ratio of median time to culture conversion on vs. off bedaquiline (0074) and the bedaquiline-associated mortality rate (0006). These results indicate that uncertainty in the optimal use of bedaquiline to improve life expectancy reflects primarily assumptions about efficacy and (possible) drug-related mortality, rather than resistance. These results are supported by the Main Text Figure 4, in which providing bedaquiline to all patients was most frequently the optimal strategy across all values of the parameters excluding culture conversion and mortality. Although their interpretation is limited to our assumed parameter distributions, they overall support the conclusion that understanding these parameters should be emphasized in future studies of bedaquiline.

SENSITIVITY TO DISTRIBUTIONAL ASSUMPTIONS

To assess the sensitivity of our results to the assumed uniform distributions, we reran the life expectancy portion of the model assuming triangular distributions with the same limits as in the Main Text Table 1 and greatest density at the midpoint. The results are shown here in Appendix Table 4. Although the simulation mean values of average life expectancy are similar to those produced by our initial uniform distributions, the bounds around these estimates are tighter, reflecting the shape of the applied triangular distributions. Furthermore, use of this distribution results in the "All MDR" strategy being optimal for a greater proportion

Table 1: Value of Information analysis results

Expected Value of Perfect Information	
Overall	0.153
Expected Value of Partial Perfect Information (BDQ Parameters)	
Default rate on BDQ	0
Relapse risk on BDQ	0
Time to culture conversion on BDQ	0.074
BDQ-related mortality	0.006
Protection from resistance to OBR	0
Rate of resistance to BDQ	0

Table 2: Life expectancy results using triangular distributions with greatest density at the midpoint. Results for average life expectancy are given as simulation mean (2.5 percentile, 97.5 percentile). Results for percent of simulations optimal are calculated out of 5,000.

	All MDR	PreXDR Only	XDR Only	None
Average Life Expectancy	36.0 (34.2, 37.9)	35.1 (34.6,35.6)	34.9 (34.7, 35.1)	34.8
Percent of Simulations Optimal	87.2	0.0	0.0	12.7

of simulations than obtained under our initial distributions, though the "None" strategy remains optimal for 12.7% of simulations.

MORTALITY SENSITIVITY ANALYSIS, RAPID SECOND-LINE DSTs

Figure 1 shows results similar to Figure 4 in the main text, except assuming the availability of rapid second-line DSTs. In this scenario, the intermediate "PreXDR+XDR" bedaquiline is more frequently preferred than when rapid second-line DSTs are unavailable.

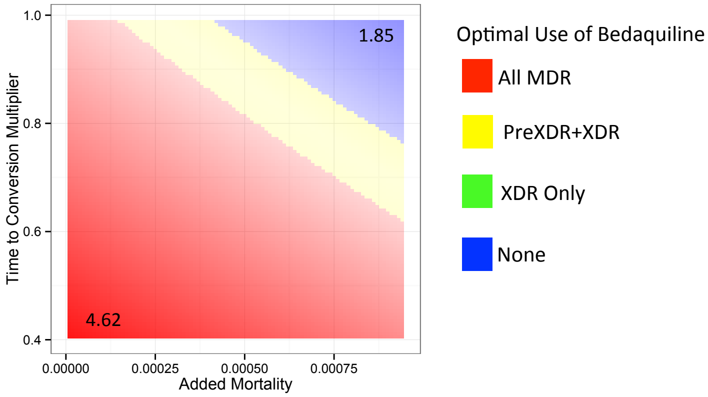

Figure 1: Heat maps showing regions in which each bedaquiline use strategy would be preferred if rapid second-line DSTs were available. The x and y axes show the explored rates of (relative) median time to culture conversion and added mortality associated with bedaquiline use. Remaining parameters are fixed at their midpoints. Colors indicate the optimal bedaquiline use strategy, and shading indicates the magnitude of difference in average life expectancy between the best and worst strategies, with the corner values listed on the figure.

ADDITIONAL ANALYSES OF XDR PLUS BEDAQUILINE RESISTANCE

To greater understand the results for our "acquired XDR plus resistance to bedaquiline" outcome, we repeated the analyses under the assumption that rapid second-line DSTs were available, as well as for different distributions of baseline resistance patterns. The results show that if rapid second-line DSTs were available, the preferred strategy would most frequently be to provide bedaquiline to all patients. Providing bedaquiline to all patients is also the most frequently preferred strategy under our baseline DST scenario when all patients are initially MDR or PreXDR. However, when all patients are initially XDR, providing bedaquiline to all patients with MDR TB is never the preferred strategy. In this case, the additional delays in starting bedaquiline prevent some patients from receiving bedaquiline (and being at risk of acquired drug resistance) prior to dying, stopping treatment, or converting to culture negative.

Table 3: Percentage of simulations for which each bedaquiline use policy minimizes acquired XDR plus resistance to bedaquiline, comparing our baseline to scenario to a rapid second-line DST scenario and different baseline resistance distributions.

	Percentage of Simulations Minimizing XDR+BDQR				
BDQ Available For	Baseline Results	Rapid DST	All Initially MDR	All Initially PreXDR	All Initially XDR
XDR Only	10.8	0.0	4.0	10.5	100.0
PreXDR+XDR	85.6	1.5	0.0	0.0	NA (same as XDR)
All MDR	3.6	98.5	96.0	89.5	0.0

Table 4: Percentage of patients acquiring XDR plus resistance to bedaquiline, comparing our baseline to scenario to a rapid second-line DST scenario and different baseline resistance distributions. Results are given as are given as simulation mean (2.5 percentile, 97.5 percentile).

	Percentage of Patients Acquiring XDR+BDQR				
BDQ Available For	Baseline Results	Rapid DST	All Initially MDR	All Initially PreXDR	All Initially XDR
XDR Only	3.5 (1.7, 5.6)	5.3 (2.0, 8.4)	0.7 (0.3, 1.1)	6.2 (2.3, 9.9)	21.2 (7.9, 34.0)
PreXDR+XDR	3.2 (1.2, 5.2)	3.7 (1.4, 6.4)	0.6 (0.2, 1.0)	5.2 (2.0, 9.2)	21.2 (7.9, 34.0)
All MDR	3.4 (1.3, 6.2)	3.4 (1.3, 6.2)	0.2 (0.1, 0.9)	4.0 (1.4, 9.6)	33.5 (12.6, 53.2)

FIXED PARAMETERS

Name	Description	Calculation & Units	Value	Explanation
Age	Age starting treatment	Years	30	
InitMDR	Proportion of people initiating MDR treatment without pre-XDR or XDR TB	Proportion	0.671	[1]
InitPreXDR	Proportion of people initiating MDR treatment with pre-XDR TB	Proportion	0.262	[1]
InitXDR	Proportion of people initiating MDR treatment with XDR TB	Proportion	0.067	[1]
MedianCCMDR	Median time to culture conversion - MDR, not on BDQ	median time in weeks, assuming no death, default, or acquired resistance	12.82 (13 allowing for acquired resistance)	[2] (13 weeks for all patients), [3] (13 weeks for MDR only), [4]
MedianCCPreXDR	Median time to culture conversion - pre-XDR, not on BDQ	median time in weeks, assuming no death, default, or acquired resistance	16.85 (18 allowing for acquired resistance)	[2] (HR 0.6 FQ-res, 0.8 2LI-res), [4]
MedianCCXDR	Median time to culture conversion - XDR, not on BDQ	median time in weeks, assuming no death, default, or acquired resistance	26	[3] (183 days), [2] (HR 0.52)
pAcquireBDQ_MDR	Weekly probability of acquiring BDQ resistance from MDR	Probability of ever acquiring BDQ resistance (initially MDR) is RiskResBDQXDR/16	See calculation page	Assuming each new drug reduces the risk of resistance 4-fold, similar to [5]
pAcquireBDQ_PreXDR	Weekly probability of acquiring BDQ resistance from pre-XDR	Probability of acquiring BDQ resistance (initially pre-XDR) is RiskResBDQXDR/4	See calculation page	[5]

pAcquireBDQ_XDR	Weekly probability of acquiring BDQ resistance from XDR	Probability of ever acquiring BDQ resistance (initially XDR) is RiskResBDQXDR	See calculation page	[5]
pAcquirePreXDR	Weekly probability of acquiring pre-XDR (from MDR, not on BDQ)	Probability of ever acquiring FQ-R or 2LI-R (initially MDR, not on BDQ) prior to death, default, stopping treatment, or conversion is 0.065 each (10.3% either)	See calculation page	[5]
pAcquirePreXDR_BDQ	Weekly probability of acquiring pre-XDR (from MDR, while on BDQ)	Probability of ever acquiring FQ-R or 2LI-R (initially MDR, on BDQ) prior to death, default, stopping treatment, or conversion is 0.065/ResProtectionBDQ	See calculation page	[5]
pAcquireXDR	Weekly probability of acquiring XDR (from pre-XDR, not on BDQ)	Probability of ever acquiring XDR (initially pre-XDR, not on BDQ) prior to death, default, stopping treatment, or conversion is 0.26	See calculation page	[5]
pAcquireXDR_BDQ	Weekly probability of acquiring XDR (from pre-XDR, while on BDQ)	Probability of ever acquiring XDR (initially pre-XDR, on BDQ) prior to death, default, stopping treatment, or conversion is 0.26/ResProtectionBDQ	See calculation page	[5]
pConvertMDR	Weekly probability of culture conversion - MDR on BR	Assuming median time to culture conversion in the absence of death, default, or acquired resistance would be MedianCCMDR	See calculation page	NA
pConvertMDR_BDQ	Weekly probability of culture conversion - MDR on BDQ	Assuming median time to culture conversion in the absence of death, default, or acquired resistance would be MedianCCMDR*BDQMedianCCRR	See calculation page	NA
pConvertPreXDR	Weekly probability of culture conversion - PreXDR on BR	Assuming median time to culture conversion in the absence of death, default, or acquired resistance would be MedianCCPreXDR	See calculation page	NA
pConvertPreXDR_BDQ	Weekly probability of culture conversion - PreXDR on BDQ	Assuming median time to culture conversion in the absence of death, default, or acquired resistance would be MedianCCPreXDR*BDQMedianCCRR	See calculation page	NA
pConvertXDR	Weekly probability of culture conversion - XDR on BR	Assuming median time to culture conversion in the absence of death, default, or acquired resistance would be MedianCCXDR	See calculation page	NA

pConvertXDR_BDQ	Weekly probability of culture conversion - XDR on BDQ	Assuming median time to culture conversion in the absence of death, default, or acquired resistance would be MedianCCXDR*BDQMedianCCRR	See calculation page	NA
pCure	Weekly probability of moving from "culture negative" (high relapse risk post-treatment) to "cure"	Reflects relapse probabilities that depend on regimen and resistance pattern	See calculation page	NA
pCureUntreated	Weekly probability of moving from "culture positive" to "cure" for untreated individuals	Assumes a case fatality rate of 60% for untreated TB	.005/(1-pDie)	[6]
pDieBDQ	Weekly probability of dying from adverse effects related to BDQ administration	Assume constant throughout treatment for all patients who have ever received BDQ, relfecting long half-life	See calculation page	NA
pDieTreatedTB	Weekly excess probability of dying from TB while culture positive on treatment	Chosen such that 16% of people with XDR on BR die prior to default or treatment completion. Excess mortality only applies while culture positive	.0075+pDieWell	[7] (15% of patients with XDR TB die, sums to 93%)
pDieUntreatedTB	Weekly excess probability of dying from TB while culture positive not on treatment	Assumed to be equal to pDieTreatedTB (case fatality of untreated TB still >> treated TB because excess mortality only applies while culture positive)	.0075+pDieWell	[7]
pDieWell	Weekly probability of dying - background mortality	Varies according to age	Taken from WHO European region male lifetables from 2013	[8]
pEnd	Probability of stopping treatment routinely	Assume average treatment duration of 21 months regardless of resistance pattern	.011/(1-pDie)	[9]
pRelapse	Weekly probability of relapse after treatment (for individuals stopping treatment not fully cured)	Assume 75% of relapses occur within one year	0.0263	[10-13]

pStartBDQPreXDR	Weekly probability of starting bedaquiline for patients with PreXDR TB receiving BR	0 if ineligible for BDQ; otherwise assume takes an average of 13 weeks for DST results<br>RDT scenario: 0 if ineligible for BDQ, 1 if eligible	0.077/((1-pDie)*(1-pDefault)*(1-pEnd))	[9]
pStartBDQXDR	Weekly probability of starting bedaquiline for patients with XDR TB receiving BR	0 if ineligible for BDQ; otherwise assume takes an average of 13 weeks for DST results<br>RDT scenario: 0 if ineligible, 1 if eligible	0.077/((1-pDie)*(1-pDefault)*(1-pEnd))	[9]
pStartTreat	Weekly probability of starting treatment for untreated, culture positive individuals	Assume average 1.5 years (78 weeks) prior to starting treatment	0.0128/((1-pDie)*(1-pCureUntreated))	[14] (1.15 years smear+, 2.39 years all confirmed TB among HIV- individuals. We have chosen an intermediate value)
pStartTreatBDQ	Probability of receiving BDQ from the initiation of MDR TB treatment	1 if all people with MDR are eligible for BDQ; 0 otherwise<br>RDT scenario: 0 if specific resistance pattern ineligible for BDQ, 1 if eligible	GetBDQMDR	NA
pStartTreatBR	Probability of receiving BR only from intiation of MDR TB treatment	0 if all people with MDR are eligible for BDQ; 1 otherwise<br>RDT scenario: 0 if specific resistance pattern eligible for BDQ, 1 if ineligible	1-GetBDQMDR	NA
RelapsePropMDR	Proportion of people with MDR TB only who ever relapse	NA	0.04	[15-17]
RelapsePropPreXDR	Proportion of people with PreXDR TB only who ever relapse	NA	0.08	[15]
RelapsePropXDR	Proportion of people with XDR TB only who ever relapse	NA	0.16	[15, 18]
RiskPreXDRBDQ	Risk of ever developing PreXDR for individuals with MDR TB receiving BDQ	Assuming BDQ reduces the risk of resistance to any fluoroquinolone or any second-line injectable by ResProtectionBDQ	See calculation page	NA

SecMultiUntreated	Ratio of infectiousness of untreated to treated individuals with culture positive TB	Assuming similar to the infectiousness of smear-positive vs. smear-negative individuals	5	[19-21]
uLE	Weekly utility when calculating life expectancy	Fraction of a year	1/52	NA
uSec_MDR	Weekly utility when calculating secondary MDR cases (on treatment, culture positive)	10 persons infected per year if smear positive*0.1 chance of progressing to active TB*.7 fitness MDR*(1/SecMultiUntreated)	0.14/52	[21-27]
uSec_MDR_BDQ	Weekly utility when calculating secondary MDR+BDQR cases (on treatment, culture positive)	10 persons infected per year if smear positive*0.1 chance of progressing to active TB*.7 fitness MDR*FitCostBDQR* (1/SecMultiUntreated)	0.14*FitCostBDQR/52	[21-27]
uSec_PreX_BDQ	Weekly utility when calculating secondary PreXDR+BDQR cases (on treatment, culture positive)	10 persons infected per year if smear positive*0.1 chance of progressing to active TB*.63 fitness PreXDRDR* FitCostBDQR* (1/SecMultiUntreated)	.126*FitCostBDQR/52	[21-27]
uSec_PreXDR	Weekly utility when calculating secondary PreXDR cases (on treatment, culture positive)	10 persons infected per year if smear positive*0.1 chance of progressing to active TB*.63 fitness PreXDR* (1/SecMultiUntreated)	.126/52	[21-27]
uSec_XDR	Weekly utility when calculating secondary XDR cases (on treatment, culture positive)	10 persons infected per year if smear positive*0.1 chance of progressing to active TB*.51 fitness XDR*(1/SecMultiUntreated)	0.113/52	[21-27]
uSec_XDR_BDQR	Weekly utility when calculating secondary XDR+BDQR cases (on treatment, culture positive)	10 persons infected per year if smear positive*0.1 chance of progressing to active TB*.51 fitness XDR*FitCostBDQR* (1/SecMultiUntreated)	0.113*FitCostBDQR/52	[21, 22, 27-30]

GetBDQMDR	Whether people with MDR TB are eligible for BDQ	Varies depending on treatment scenario	1 if yes, 0 if no	NA
GetBDQPreXDR	Whether people with PreXDR TB are eligible for BDQ	Varies depending on treatment scenario	1 if yes, 0 if no	NA
GetBDQXDR	Whether people with XDR TB are eligible for BDQ	Varies depending on treatment scenario	1 if yes, 0 if no	NA
pDefault	Weekly default probability	Constant throughout treatment, value depends on regimen. Baseline is chosen such that 17.3% of people with XDR on BR default prior to death or stopping treatment.	See TreeAge file	[7]
pDie	Weekly probability of death	Depends on whether the individual is culture positive or receiving bedaquiline and their age	Sum of relevant probabilities	NA
RelapseProp	Proportion of people who would relapse post-treatment	Depends on the individual's regimen and resistance pattern	See TreeAge file	NA

COMPLETE LIST OF MODEL TRANSITIONS

From States Receiving Bedaquiline

MDR, Bedaquiline-Sensitive, On Bedaquiline, Culture+

Die -> Death

Stop treatment (routine) -> MDR, Bedaquiline-Sensitive, Not on Treatment, Culture+

Default -> MDR, Bedaquiline-Sensitive, Not on Treatment, Culture+

Convert -> MDR, Bedaquiline-Sensitive, On Bedaquiline, Culture-

Acquire PreXDR -> PreXDR, Bedaquiline-Sensitive, On Bedaquiline, Culture+

Acquire BDQR -> MDR, Bedaquiline-Resistant, On Bedaquiline, Culture+

Remain in same state

MDR, Bedaquiline-Resistant, On Bedaquiline, Culture+

Die -> Death

Stop treatment (routine) -> MDR, Bedaquiline-Resistant, Not on Treatment, Culture+

Default -> MDR, Bedaquiline-Resistant, Not on Treatment, Culture+

Convert -> MDR, Bedaquiline-Resistant, On Bedaquiline, Culture-

Acquire PreXDR -> PreXDR, Bedaquiline-Resistant, On Bedaquiline, Culture+

Remain in same state

MDR, Bedaquiline-Sensitive, On Bedaquiline, Culture-

Die -> Death

Stop treatment (routine) -> MDR, Bedaquiline-Sensitive, Not on Treatment, Culture-

Default -> MDR, Bedaquiline-Sensitive, Not on Treatment, Culture-

Cure -> Any Resistance, On Bedaquiline, Cured

Remain in same state

MDR, Bedaquiline-Resistant, On Bedaquiline, Culture-

Die -> Death

Stop treatment (routine) -> MDR, Bedaquiline-Resistant, Not on Treatment, Culture-

Default -> MDR, Bedaquiline-Resistant, Not on Treatment, Culture-

Cure -> Any Resistance, On Bedaquiline, Cured

Remain in same state

PreXDR, Bedaquiline-Sensitive, On Bedaquiline, Culture+

Die -> Death

Stop treatment (routine) -> PreXDR, Bedaquiline-Sensitive, Not on Treatment, Culture+

Default -> PreXDR, Bedaquiline-Sensitive, Not on Treatment, Culture+

Convert -> PreXDR, Bedaquiline-Sensitive, On Bedaquiline, Culture-

Acquire XDR -> XDR, Bedaquiline-Sensitive, On Bedaquiline, Culture+

Acquire BDQR -> PreXDR, Bedaquiline-Resistant, On Bedaquiline, Culture+

Remain in same state

PreXDR, Bedaquiline-Resistant, On Bedaquiline, Culture+

Die -> Death

Stop treatment (routine) -> PreXDR, Bedaquiline-Resistant, Not on Treatment, Culture+

Default -> PreXDR, Bedaquiline-Resistant, Not on Treatment, Culture+

Convert -> PreXDR, Bedaquiline-Resistant, On Bedaquiline, Culture-

Acquire XDR -> XDR, Bedaquiline-Resistant, On Bedaquiline, Culture+

Remain in same state

PreXDR, Bedaquiline-Sensitive, On Bedaquiline, Culture-

Die -> Death

Stop treatment (routine) -> PreXDR, Bedaquiline-Sensitive, Not on Treatment, Culture-

Default -> PreXDR, Bedaquiline-Sensitive, Not on Treatment, Culture-

Cure -> Any Resistance, On Bedaquiline, Cured

Remain in same state

PreXDR, Bedaquiline-Resistant, On Bedaquiline, Culture-

Die -> Death

Stop treatment (routine) -> PreXDR, Bedaquiline-Resistant, Not on Treatment, Culture-

Default -> PreXDR, Bedaquiline-Resistant, Not on Treatment, Culture-

Cure -> Any Resistance, On Bedaquiline, Cured

Remain in same state

XDR, Bedaquiline-Sensitive, On Bedaquiline, Culture+

Die -> Death

Stop treatment (routine) -> XDR, Bedaquiline-Sensitive, Not on Treatment, Culture+

Default -> XDR, Bedaquiline-Sensitive, Not on Treatment, Culture+

Convert -> XDR, Bedaquiline-Sensitive, On Bedaquiline, Culture-

Acquire BDQR -> XDR, Bedaquiline-Resistant, On Bedaquiline, Culture+

Remain in same state

XDR, Bedaquiline-Resistant, On Bedaquiline, Culture+

Die -> Death

Stop treatment (routine) -> XDR, Bedaquiline-Resistant, Not on Treatment, Culture+

Default -> XDR, Bedaquiline-Resistant, Not on Treatment, Culture+

Convert -> XDR, Bedaquiline-Resistant, On Bedaquiline, Culture-

Remain in same state

XDR, Bedaquiline-Sensitive, On Bedaquiline, Culture-

Die -> Death

Stop treatment (routine) -> XDR, Bedaquiline-Sensitive, Not on Treatment, Culture-

Default -> XDR, Bedaquiline-Sensitive, Not on Treatment, Culture-

Cure -> Any Resistance, On Bedaquiline, Cured

Remain in same state

XDR, Bedaquiline-Resistant, On Bedaquiline, Culture-

Die -> Death

Stop treatment (routine) -> XDR, Bedaquiline-Resistant, Not on Treatment, Culture-

Default -> XDR, Bedaquiline-Resistant, Not on Treatment, Culture-

Cure -> Any Resistance, On Bedaquiline, Cured

Remain in same state

Any Resistance, On Bedaquiline, Cured

Die -> Death

Stop treatment (routine) -> Any Resistance, Not on Treatment, Cured

Default -> Any Resistance, Not on Treatment, Cured

Remain in same state

From States Receiving OBR

MDR, Bedaquiline-Sensitive, On OBR, Culture+

Die -> Death

Stop treatment (routine) -> MDR, Bedaquiline-Sensitive, Not on Treatment, Culture+

Default -> MDR, Bedaquiline-Sensitive, Not on Treatment, Culture+

Convert -> MDR, Bedaquiline-Sensitive, On OBR, Culture-

Acquire PreXDR -> PreXDR, Bedaquiline-Sensitive, On OBR, Culture+

Remain in same state

MDR, Bedaquiline-Resistant, On OBR, Culture+

Die -> Death

Stop treatment (routine) -> MDR, Bedaquiline-Resistant, Not on Treatment, Culture+

Default -> MDR, Bedaquiline-Resistant, Not on Treatment, Culture+

Convert -> MDR, Bedaquiline-Resistant, On OBR, Culture-

Acquire PreXDR -> PreXDR, Bedaquiline-Resistant, On OBR, Culture+

Remain in same state

MDR, Bedaquiline-Sensitive, On OBR, Culture-

Die -> Death

Stop treatment (routine) -> MDR, Bedaquiline-Sensitive, Not on Treatment, Culture-

Default -> MDR, Bedaquiline-Sensitive, Not on Treatment, Culture-

Cure -> MDR, On OBR, Cured

Remain in same state

MDR, Bedaquiline-Resistant, On OBR, Culture-

Die -> Death

Stop treatment (routine) -> MDR, Bedaquiline-Resistant, Not on Treatment, Culture-

Default -> MDR, Bedaquiline-Resistant, Not on Treatment, Culture-

Cure -> MDR, On OBR, Cured

Remain in same state

PreXDR, Bedaquiline-Sensitive, On OBR, Culture+

Die -> Death

Stop treatment (routine) -> PreXDR, Bedaquiline-Sensitive, Not on Treatment, Culture+

Default -> PreXDR, Bedaquiline-Sensitive, Not on Treatment, Culture+

Convert & Start BDQ -> PreXDR, Bedaquiline-Sensitive, On Bedaquiline, Culture-

Convert & Continue OBR -> PreXDR, Bedaquiline-Sensitive, On OBR, Culture-

Acquire XDR & Start BDQ -> XDR, Bedaquiline-Sensitive, On Bedaquiline, Culture+

Acquire XDR & Continue OBR -> XDR, Bedaquiline-Sensitive, On OBR, Culture+

Start BDQ -> PreXDR, Bedaquiline-Sensitive, On Bedaquiline, Culture+

Remain in same state

PreXDR, Bedaquiline-Resistant, On OBR, Culture+

Die -> Death

Stop treatment (routine) -> PreXDR, Bedaquiline-Resistant, Not on Treatment, Culture+

Default -> PreXDR, Bedaquiline-Resistant, Not on Treatment, Culture+

Convert & Start BDQ -> PreXDR, Bedaquiline-Resistant, On Bedaquiline, Culture-

Convert & Continue OBR -> PreXDR, Bedaquiline-Resistant, On OBR, Culture-

Acquire XDR & Start BDQ -> XDR, Bedaquiline-Resistant, On Bedaquiline, Culture+

Acquire XDR & Continue OBR -> XDR, Bedaquiline-Resistant, On OBR, Culture+

Start BDQ -> PreXDR, Bedaquiline-Resistant, On Bedaquiline, Culture+

Remain in same state

PreXDR, Bedaquiline-Sensitive, On OBR, Culture-

Die -> Death

Stop treatment (routine) -> PreXDR, Bedaquiline-Sensitive, Not on Treatment, Culture-

Default -> PreXDR, Bedaquiline-Sensitive, Not on Treatment, Culture-

Cure & Start BDQ -> Any Resistance, On Bedaquiline, Cured

Cure & Continue OBR -> PreXDR, On OBR, Cured

Start BDQ -> PreXDR, Bedaquiline-Sensitive, On Bedaquiline, Culture-

Remain in same state

PreXDR, Bedaquiline-Resistant, On OBR, Culture-

Die -> Death

Stop treatment (routine) -> PreXDR, Bedaquiline-Resistant, Not on Treatment, Culture-

Default -> PreXDR, Bedaquiline-Resistant, Not on Treatment, Culture-

Cure & Start BDQ -> Any Resistance, On Bedaquiline, Cured

Cure & Continue OBR -> PreXDR, On OBR, Cured

Start BDQ -> PreXDR, Bedaquiline-Resistant, On Bedaquiline, Culture-

Remain in same state

XDR, Bedaquiline-Sensitive, On OBR, Culture+

Die -> Death

Stop treatment (routine) -> XDR, Bedaquiline-Sensitive, Not on Treatment, Culture+

Default -> XDR, Bedaquiline-Sensitive, Not on Treatment, Culture+

Convert & Start BDQ -> XDR, Bedaquiline-Sensitive, On Bedaquiline, Culture-

Convert & Continue OBR -> XDR, Bedaquiline-Sensitive, On OBR, Culture-

Start BDQ -> XDR, Bedaquiline-Sensitive, On Bedaquiline, Culture+

Remain in same state

XDR, Bedaquiline-Resistant, On OBR, Culture+

Die -> Death

Stop treatment (routine) -> XDR, Bedaquiline-Resistant, Not on Treatment, Culture+

Default -> XDR, Bedaquiline-Resistant, Not on Treatment, Culture+

Convert & Start BDQ -> XDR, Bedaquiline-Resistant, On Bedaquiline, Culture-

Convert & Continue OBR -> XDR, Bedaquiline-Resistant, On OBR, Culture-

Start BDQ -> XDR, Bedaquiline-Resistant, On Bedaquiline, Culture+

Remain in same state

XDR, Bedaquiline-Sensitive, On OBR, Culture-

Die -> Death

Stop treatment (routine) -> XDR, Bedaquiline-Sensitive, Not on Treatment, Culture-

Default -> XDR, Bedaquiline-Sensitive, Not on Treatment, Culture-

Cure & Start BDQ -> Any Resistance, On Bedaquiline, Cured

Cure & Continue OBR -> XDR, On OBR, Cured

Start BDQ -> XDR, Bedaquiline-Sensitive, On Bedaquiline, Culture-

Remain in same state

XDR, Bedaquiline-Resistant, On OBR, Culture-

Die -> Death

Stop treatment (routine) -> XDR, Bedaquiline-Resistant, Not on Treatment, Culture-

Default -> XDR, Bedaquiline-Resistant, Not on Treatment, Culture-

Cure & Start BDQ -> Any Resistance, On Bedaquiline, Cured

Cure & Continue OBR -> XDR, On OBR, Cured

Start BDQ -> XDR, Bedaquiline-Resistant, On Bedaquiline, Culture-

Remain in same state

MDR, On OBR, Cured

Die -> Death

Stop treatment (routine) -> Any Resistance, Not on Treatment, Cured

Default -> Any Resistance, Not on Treatment, Cured

Remain in same state

PreXDR, On OBR, Cured

Die -> Death

Stop treatment (routine) -> Any Resistance, Not on Treatment, Cured

Default -> Any Resistance, Not on Treatment, Cured

Start BDQ -> Any Resistance, On Bedaquiline, Cured

Remain in same state

XDR, On OBR, Cured

Die -> Death

Stop treatment (routine) -> Any Resistance, Not on Treatment, Cured

Default -> Any Resistance, Not on Treatment, Cured

Start BDQ -> Any Resistance, On Bedaquiline, Cured

Remain in same state

From States Not on Treatment

MDR, Bedaquiline-Sensitive, Not on Treatment, Culture+

Die -> Death

Cure -> Any Resistance, Not on Treatment, Cured

Start treatment with OBR -> MDR, Bedaquiline-Sensitive, On OBR, Culture+

Start treatment with BDQ -> MDR, Bedaquiline-Sensitive, On Bedaquiline, Culture+

Remain in same state

MDR, Bedaquiline-Resistant, Not on Treatment, Culture+

Die -> Death

Cure -> Any Resistance, Not on Treatment, Cured

Start treatment with OBR -> MDR, Bedaquiline-Resistant, On OBR, Culture+

Start treatment with BDQ -> MDR, Bedaquiline-Resistant, On Bedaquiline, Culture+

Remain in same state

MDR, Bedaquiline-Sensitive, Not on Treatment, Culture-

Die -> Death

Relapse -> MDR, Bedaquiline-Sensitive, Not on Treatment, Culture+

Remain in same state

MDR, Bedaquiline-Resistant, Not on Treatment, Culture-

Die -> Death

Relapse -> MDR, Bedaquiline-Resistant, Not on Treatment, Culture+

Remain in same state

PreXDR, Bedaquiline-Sensitive, Not on Treatment, Culture+

Die -> Death

Cure -> Any Resistance, Not on Treatment, Cured

Start treatment with OBR -> PreXDR, Bedaquiline-Sensitive, On OBR, Culture+

Start treatment with BDQ -> PreXDR, Bedaquiline-Sensitive, On Bedaquiline, Culture+

Remain in same state

PreXDR, Bedaquiline-Resistant, Not on Treatment, Culture+

Die -> Death

Cure -> Any Resistance, Not on Treatment, Cured

Start treatment with OBR -> PreXDR, Bedaquiline-Resistant, On OBR, Culture+

Start treatment with BDQ -> PreXDR, Bedaquiline-Resistant, On Bedaquiline, Culture+

Remain in same state

PreXDR, Bedaquiline-Sensitive, Not on Treatment, Culture-

Die -> Death

Relapse -> PreXDR, Bedaquiline-Sensitive, Not on Treatment, Culture+

Remain in same state

PreXDR, Bedaquiline-Resistant, Not on Treatment, Culture-

Die -> Death

Relapse -> PreXDR, Bedaquiline-Resistant, Not on Treatment, Culture+

Remain in same state

XDR, Bedaquiline-Sensitive, Not on Treatment, Culture+

Die -> Death

Cure -> Any Resistance, Not on Treatment, Cured

Start treatment with OBR -> XDR, Bedaquiline-Sensitive, On OBR, Culture+

Start treatment with BDQ -> XDR, Bedaquiline-Sensitive, On Bedaquiline, Culture+

Remain in same state

XDR, Bedaquiline-Resistant, Not on Treatment, Culture+

Die -> Death

Cure -> Any Resistance, Not on Treatment, Cured

Start treatment with OBR -> XDR, Bedaquiline-Resistant, On OBR, Culture+

Start treatment with BDQ -> XDR, Bedaquiline-Resistant, On Bedaquiline, Culture+

Remain in same state

XDR, Bedaquiline-Sensitive, Not on Treatment, Culture-

Die -> Death

Relapse -> XDR, Bedaquiline-Sensitive, Not on Treatment, Culture+

Remain in same state

XDR, Bedaquiline-Resistant, Not on Treatment, Culture-

Die -> Death

Relapse -> XDR, Bedaquiline-Resistant, Not on Treatment, Culture+

Remain in same state

Any Resistance, Not on Treatment, Cured

Die -> Death

Remain in same state

Death

Remain in same state

REFERENCES

1. Dalton T, Cegielski P, Akksilp S, Asencios L, Campos Caoili J, Cho SN et al. Prevalence of and risk factors for resistance to second-line drugs in people with multidrug-resistant tuberculosis in eight countries: a prospective cohort study. Lancet. 2012;380(9851):1406-17. doi:10.1016/s0140-6736(12)60734-x.
2. Kurbatova E, Gammino VM, Bayona J, Becerra MC, Danilovitz M, Falzon D et al. Predictors of sputum culture conversion among patients treated for multidrug-resistant tuberculosis. International Journal of Tuberculosis and Lung Disease. 2012;16:1335-43. doi:10.5588/ijtld.11.0811.
3. Shah NS, Pratt R, Armstrong L, Robison V, Castro KG, Cegielski JP. Extensively drug-resistant tuberculosis in the United States, 1993-2007. Jama. 2008;300(18):2153-60. doi:10.1001/jama.300.18.2153.
4. Diacon AH, Lounis N, Dannemann B. Multidrug-resistant tuberculosis and bedaquiline. The New England journal of medicine. 2014;371(25):2436. doi:10.1056/NEJMc1412235.
5. Cegielski JP, Dalton T, Yagui M, Wattanaamornkiet W, Volchenkov GV, Via LE et al. Extensive drug resistance acquired during treatment of multidrug-resistant tuberculosis. Clinical infectious diseases : an official publication of the Infectious Diseases Society of America. 2014;59(8):1049-63. doi:10.1093/cid/ciu572.
6. Tiemersma EW, van der Werf MJ, Borgdorff MW, Williams BG, Nagelkerke NJD. Natural history of tuberculosis: duration and fatality of untreated pulmonary tuberculosis in HIV negative patients: a systematic review. PloS one. 2011;6:e17601. doi:10.1371/journal.pone.0017601.
7. Falzon D, Gandhi N, Migliori GB, Sotgiu G, Cox H, Holtz TH et al. Resistance to fluoroquinolones and second-line injectable drugs: impact on MDR-TB outcomes. The European respiratory journal : official journal of the European Society for Clinical Respiratory Physiology. 2012:1-24. doi:10.1183/09031936.00134712.
8. World Health Organization. Life tables by WHO region: Europe. In: Global Health Observatory data repository. 2015. Accessed June 11 2015.
9. World Health Organization. Guidelines for the programmatic management of drug-resistant tuberculosis: 2011 update. Geneva2011.
10. Glynn JR, Murray J, Bester A, Nelson G, Shearer S, Sonnenberg P. High rates of recurrence in HIV-infected and HIV-uninfected patients with tuberculosis. The Journal of infectious diseases. 2010;201(5):704-11. doi:10.1086/650529.

11. Guerra-Assuncao JA, Houben RM, Crampin AC, Mzembe T, Mallard K, Coll F et al. Recurrence due to relapse or reinfection with Mycobacterium tuberculosis: a whole-genome sequencing approach in a large, population-based cohort with a high HIV infection prevalence and active follow-up. The Journal of infectious diseases. 2015;211(7):1154-63. doi:10.1093/infdis/jiu574.
12. Marx FM, Dunbar R, Enarson DA, Williams BG, Warren RM, van der Spuy GD et al. The temporal dynamics of relapse and reinfection tuberculosis after successful treatment: a retrospective cohort study. Clinical infectious diseases : an official publication of the Infectious Diseases Society of America. 2014;58(12):1676-83. doi:10.1093/cid/ciu186.
13. Nunn AJ, Phillips PP, Mitchison DA. Timing of relapse in short-course chemotherapy trials for tuberculosis. The international journal of tuberculosis and lung disease : the official journal of the International Union against Tuberculosis and Lung Disease. 2010;14(2):241-2.
14. Corbett EL, Charalambous S, Moloi VM, Fielding K, Grant AD, Dye C et al. Human immunodeficiency virus and the prevalence of undiagnosed tuberculosis in African gold miners. American journal of respiratory and critical care medicine. 2004;170(6):673-9. doi:10.1164/rccm.200405-590OC.
15. Chiang CY, Enarson DA, Yu MC, Bai KJ, Huang RM, Hsu CJ et al. Outcome of pulmonary multidrug-resistant tuberculosis: a 6-yr follow-up study. The European respiratory journal. 2006;28(5):980-5. doi:10.1183/09031936.06.00125705.
16. Johnston JC, Khan FA, Dowdy DW. Reducing relapse in tuberculosis treatment: is it time to reassess WHO treatment guidelines? The international journal of tuberculosis and lung disease : the official journal of the International Union against Tuberculosis and Lung Disease. 2015;19(6):624. doi:10.5588/ijtld.15.0224.
17. Gelmanova IY, Ahmad Khan F, Becerra MC, Zemlyanaya NA, Unakova IA, Andreev YG et al. Low rates of recurrence after successful treatment of multidrug-resistant tuberculosis in Tomsk, Russia. The international journal of tuberculosis and lung disease : the official journal of the International Union against Tuberculosis and Lung Disease. 2015;19(4):399-405. doi:10.5588/ijtld.14.0415.
18. Menzies D, Benedetti A, Paydar A, Martin I, Royce S, Pai M et al. Effect of duration and intermittency of rifampin on tuberculosis treatment outcomes: a systematic review and meta-analysis. PLoS medicine. 2009;6(9):e1000146. doi:10.1371/journal.pmed.1000146.
19. Tostmann A, Kik SV, Kalisvaart NA, Sebek MM, Verver S, Boeree MJ et al. Tuberculosis transmission by patients with smear-negative pulmonary tuberculosis in a large cohort in the Netherlands. Clinical infectious diseases : an official publication of the Infectious Diseases Society of America. 2008;47(9):1135-42. doi:10.1086/591974.
20. Behr MA, Warren SA, Salamon H, Hopewell PC, Ponce de Leon A, Daley CL et al. Transmission of Mycobacterium tuberculosis from patients smear-negative for acid-fast bacilli. Lancet. 1999;353(9151):444-9.
21. Styblo K. Epidemiology of Tuberculosis: Selected Papers. The Hague: Royal Netherlands Tuberculosis Foundation (KNCV); 1991.
22. Trunz BB, Fine P, Dye C. Effect of BCG vaccination on childhood tuberculous meningitis and miliary tuberculosis worldwide: a meta-analysis and assessment of cost-effectiveness. Lancet. 2006;367(9517):1173-80. doi:10.1016/s0140-6736(06)68507-3.
23. Gagneux S, Long CD, Small PM, Van T, Schoolnik GK, Bohannan BJ. The competitive cost of antibiotic resistance in Mycobacterium tuberculosis. Science (New York, NY). 2006;312(5782):1944-6. doi:10.1126/science.1124410.
24. Luciani F, Sisson SA, Jiang H, Francis AR, Tanaka MM. The epidemiological fitness cost of drug resistance in Mycobacterium tuberculosis. Proceedings of the National Academy of Sciences of the United States of America. 2009;106(34):14711-5. doi:10.1073/pnas.0902437106.
25. Bottger EC, Springer B. Tuberculosis: drug resistance, fitness, and strategies for global control. European journal of pediatrics. 2008;167(2):141-8. doi:10.1007/s00431-007-0606-9.
26. Grandjean L, Gilman RH, Martin L, Soto E, Castro B, Lopez S et al. Transmission of Multidrug-Resistant and Drug-Susceptible Tuberculosis within Households: A Prospective Cohort Study. PLoS medicine. 2015;12(6):e1001843; discussion e. doi:10.1371/journal.pmed.1001843.
27. Vynnycky E, Fine PE. The natural history of tuberculosis: the implications of age-dependent risks of disease and the role of reinfection. Epidemiology and infection. 1997;119(2):183-201.
28. Becerra MC, Appleton SC, Franke MF, Chalco K, Arteaga F, Bayona J et al. Tuberculosis burden in households of patients with multidrug-resistant and extensively drug-resistant tuberculosis: a retrospective cohort study. Lancet. 2011;377(9760):147-52. doi:10.1016/s0140-6736(10)61972-1.

29. Naidoo CC, Pillay M. Increased in vitro fitness of multi- and extensively drug-resistant F15/LAM4/KZN strains of Mycobacterium tuberculosis. Clinical microbiology and infection : the official publication of the European Society of Clinical Microbiology and Infectious Diseases. 2014;20(6):O361-9. doi:10.1111/1469-0691.12415.
30. Smith KL, Saini D, Bardarov S, Larsen M, Frothingham R, Gandhi NR et al. Reduced virulence of an extensively drug-resistant outbreak strain of Mycobacterium tuberculosis in a murine model. PloS one. 2014;9(4):e94953. doi:10.1371/journal.pone.0094953.
